# Health assessment of nesting loggerhead sea turtles (*Caretta caretta*) in one of their largest rookeries (central eastern Florida coast, USA)

**DOI:** 10.1093/conphys/coae064

**Published:** 2024-09-20

**Authors:** Nicole I Stacy, Rachel Smith, Kathleen E Sullivan, Steven E Nelson Jr, Elizabeth C Nolan, Ryan S De Voe, Blair E Witherington, Justin R Perrault

**Affiliations:** Department of Large Animal Clinical Sciences, College of Veterinary Medicine, University of Florida, 2015 SW 16TH AVE, Gainesville, FL 32610, USA; Disney’s Animals, Science and Environment, 1200 N Savannah Circle, Disney's Animal Kingdom, Lake Buena Vista, FL 32830, USA; Disney’s Animals, Science and Environment, 1200 N Savannah Circle, Disney's Animal Kingdom, Lake Buena Vista, FL 32830, USA; Disney’s Animals, Science and Environment, 1200 N Savannah Circle, Disney's Animal Kingdom, Lake Buena Vista, FL 32830, USA; Disney’s Animals, Science and Environment, 1200 N Savannah Circle, Disney's Animal Kingdom, Lake Buena Vista, FL 32830, USA; Disney’s Animals, Science and Environment, 1200 N Savannah Circle, Disney's Animal Kingdom, Lake Buena Vista, FL 32830, USA; Disney’s Animals, Science and Environment, 1200 N Savannah Circle, Disney's Animal Kingdom, Lake Buena Vista, FL 32830, USA; Disney’s Animals, Science and Environment, 1200 N Savannah Circle, Disney's Animal Kingdom, Lake Buena Vista, FL 32830, USA; Inwater Research Group, Inc., 4160 NE Hyline Dr, Jensen Beach, FL 34957, USA; Loggerhead Marinelife Center, 14200 US Highway 1, Juno Beach, FL 33408, USA

**Keywords:** Blood analysis, body condition index, fatty acids, haematology, morphometrics, plasma biochemistry, protein electrophoresis, reference intervals, trace nutrients, vitamins

## Abstract

Reproduction is a physiologically demanding process for sea turtles. Health indicators, including morphometric indices and blood analytes, provide insight into overall health, physiology and organ function for breeding sea turtles as a way to assess population-level effects. The Archie Carr National Wildlife Refuge (ACNWR) on Florida’s central eastern coast is critical nesting habitat for loggerhead sea turtles (*Caretta caretta*), but health variables from this location have not been documented. Objectives of the study were to (1) assess morphometrics and blood analyte data (including haematology, plasma biochemistry, protein electrophoresis, β-hydroxybutyrate, trace nutrients, vitamins and fatty acid profiles) from loggerheads nesting on or near the beaches of the ACNWR, (2) investigate correlations of body condition index (BCI) with blood analytes and (3) analyse temporal trends in morphometric and blood analyte data throughout the nesting season. Morphometric and/or blood analyte data are reported for 57 nesting loggerheads encountered between 2016 and 2019. Plasma copper and iron positively correlated with BCI. Mass tended to decline across nesting season, whereas BCI did not. Many blood analytes significantly increased or decreased across nesting season, reflecting the catabolic state and haemodynamic variations of nesting turtles. Twenty-three of 34 fatty acids declined across nesting season, which demonstrates the physiological demands of nesting turtles for vitellogenesis and reproductive activities, thus suggesting potential utility of fatty acids for the assessment of foraging status and phases of reproduction. The findings herein are relevant for future spatiotemporal and interspecies comparisons, investigating stressor effects and understanding the physiological demands in nesting sea turtles. This information provides comparative data for individual animals in rescue or managed care settings and for assessment of conservation strategies.

## Introduction

Loggerhead sea turtles (*Caretta caretta*) are protected under the Convention on International Trade in Endangered Species Appendix I as well as the US Endangered Species Act and listed as vulnerable on the red list of the International Union for the Conservation of Nature (Casale & Tucker, 2017; IUCN 2024). Similar to other sea turtle species worldwide, loggerheads face various threats, including fisheries bycatch, climate change, boating interactions, illegal hunting, nesting beach loss or degradation, pollution and disease (Ataman *et al.,* 2021; Bolten *et al.*, 2011; Bolten *et al.*, 2019; Donlan *et al.*, 2010; Foley *et al.*, 2019; Fuentes *et al.*, 2016, Fuentes *et al.*, 2023; Page-Karjian *et al.*, 2020; Wallace *et al.*, 2010). Major threats for eggs and hatchlings include pollution (including artificial lighting), beach degradation (including coastal armoring), increasing nest temperatures and predation (Bolten *et al.*, 2011).

Adult female sea turtles experience physiological demands during their reproductive migration and nesting season. Loggerheads migrate hundreds of kilometres to their nesting beaches and lay 4–8 clutches at an inter-nesting interval of 10–19 days, with a remigration interval of 2–5 years (Hirsch, 2022; Miller, 1997; Tucker, 2010). The largest aggregations of nesting loggerheads worldwide are found on the central and southeastern Atlantic coast of Florida and on eastern Masirah Island in the Sultanate of Oman (Ataman *et al.*, 2021; Casale & Tucker, 2017; Ceriani *et al.*, 2017; Ceriani *et al.*, 2019; Ceriani & Meylan, 2017; Ehrhart *et al.*, 2014; Hirsch, 2022; Willson *et al.*, 2020). Considered one of the most important regions for loggerhead nesting worldwide, the Archie Carr National Wildlife Refuge (ACNWR) is located along the beaches of central eastern Florida. Between 2014 and 2023, ACNWR beaches supported an annual average of 12 733 ± 2279 loggerhead nests along 21 km of protected coastline (personal communication from Dr E. Seney, University of Central Florida). The loggerhead population nesting in the ACNWR is part of the Northwest Atlantic Regional Management Unit, currently a stable population, but at risk of population decline if current threats remain unabated (Ceriani *et al.*, 2017; Wallace *et al.*, 2010). Loggerheads appear to have high site fidelity to nesting beaches, and although they may re-nest at a range of up to 290 km within one nesting season; however, intraannual nesting ranges are typically <10 km (Bjorndal *et al.*, 1983; Hirsch, 2022; Tucker, 2010; Weishampel *et al.*, 2003). This site fidelity is thought to divide Florida nesting loggerheads amongst four genetically distinct management populations, within which ACNWR turtles occupy a central eastern Florida group (Shamblin *et al.*, 2011).

In addition to the high physiological demands of reproductive activities (e.g. migration, vitellogenesis, nesting), nesting loggerheads undergo times of hyporexia as part of a capital breeding strategy, which adds to their metabolic challenges during this phase of their life cycle (Perrault & Stacy, 2018). These characteristics highlight the importance of long-term health monitoring in sea turtles to better understand their populations and the value of health surveillance data to inform policymakers (Fuentes *et al.*, 2023; Page-Karjian & Perrault, 2021).

To answer specific questions regarding population health, health assessment studies in wildlife species aim to collect targeted samples from live animals in a way that minimizes effects from sampling on the animals’ behavioural and physiological states. Establishment of baseline health variables for loggerheads is considered a priority action in the most recent recovery plan for the Northwest Atlantic population of loggerheads (Fuentes *et al.*, 2023; Hamann *et al.*, 2010; Bolten *et al*., 2019; NMFS & USFWS, 2008).

Blood is a sample matrix that can be used to gain information on many health variables. Sampling blood is minimally invasive and causes low risk of disturbing the nesting process if performed per standardized recommendations (NMFS SEFSC, 2008). Establishment of blood analyte baselines for various sea turtle species from different geographical sites and life-stage classes allows a better understanding of population health, is important for evaluating impacts of stressors on the population and may be useful for improving rehabilitation outcomes and animal welfare measures. For these reasons, blood analyte data have been reported for many sea turtle species, life-stage classes, from various geographical areas and in different settings (Aguirre & Balazs, 2000; Fleming *et al.*, 2020; Flint *et al.*, 2010, Flint *et al.*, 2015; Kelly *et al.*, 2015; Osborne *et al.*, 2010; Page-Karjian *et al.*, 2015, Page-Karjian *et al.*, 2020; Perrault *et al.*, 2014, Perrault *et al.*, 2020, Perrault *et al.*, 2022; Stacy & Innis 2017; Stacy *et al.*, 2018, Stacy *et al.*, 2019, Stacy *et al.*, 2023). However, blood data from nesting loggerheads at Florida’s central eastern coast have not been published to date.

**Figure 1:**
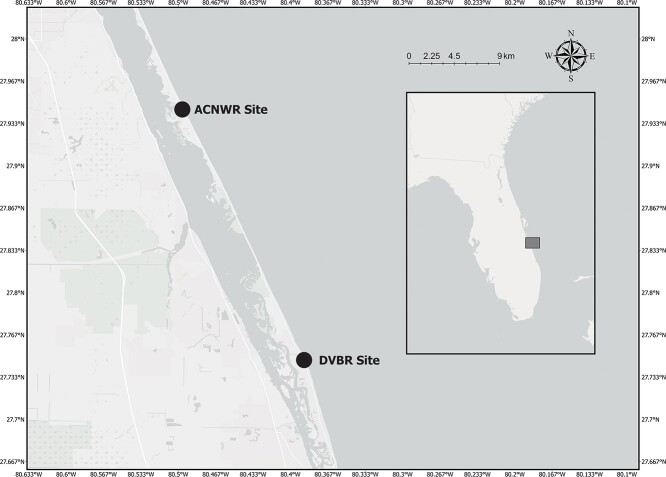
Locations of both sampling sites of loggerhead sea turtles (*Caretta caretta*) at the ACNWR, Florida, USA.

Nesting females represent an important life stage for collecting health data given their unique physiological state during phases of active reproduction and their value in maintaining the population (Goldberg *et al.*, 2013; Hays *et al.*, 2002; Honarvar *et al.*, 2011; Page-Karjian *et al.*, 2020; Perrault *et al.*, 2012, Perrault *et al.*, 2014, Perrault *et al.*, 2016; Perrault & Stacy, 2018; Plot *et al.*, 2013; Smith, 2010). Health variables of nesting females, including morphometric data and blood analyses related to metabolism and nutrition, provide valuable insight into physiological changes associated with capital breeding during nesting seasons and for geographical variations amongst populations. The objectives of this study were to (1) assess morphometrics and comprehensive blood analyte data (including haematology, routine plasma biochemistry, protein electrophoresis, β-hydroxybutyrate [BHB], trace nutrients, vitamins and fatty acid profiles) from loggerheads nesting on or near the beaches of the ACNWR, (2) investigate correlations of body condition index (BCI) with blood analytes and (3) analyse temporal trends in morphometric and blood analyte data across nesting season.

## Materials and Methods

### Ethical procedures

This research was conducted under Florida Fish & Wildlife Conservation Commission Marine Turtle Permit 071 and USFWS NWR Permit No. 4157520167. Scientific merit and animal welfare reviews were completed through an internal review process by Disney’s Animal Care and Welfare Committee (DACWC #IR 1604).

### Study site

Loggerheads nesting within or near the ACNWR in Brevard and Indian River Counties, Florida, USA were sampled for this study (Fig. 1). Loggerheads nesting in Brevard County were sampled on the beach between 27.9536°N, −80.5012°W and 27.9296°N, −80.4867°W. Samples from loggerheads in Indian River County, Disney’s Vero Beach Resort were collected on the beach between 27.7679°N, −80.3988°W and 27.7448°N, −80.3860°W, ~2 km south of the ACNWR.

### Blood sampling and morphometric measurements

Trained researchers patrolled the ACNWR on foot at night, between 2100 and 0400 h, May–August 2017–19 (with one pilot trip in August 2016). Turtles were not approached until they entered the laying phase of nesting, when they are minimally responsive to external stimuli and unlikely to abandon nesting (i.e. nesting fixed action pattern) (Hailman & Elowson, 1992; Smith *et al.,* 2021). Blood collection during oviposition also ensured safe blood draws without manual restraint, as turtles are immobile during this phase of nesting. The venipuncture site (external jugular vein) was cleaned of debris with fresh water, followed by three alternating repetitions of 0.4% chlorhexidine gluconate solution (Phoenix™, Elgin, IL) and 70% isopropyl alcohol (Aspen Veterinary Resources, LTD., Greeley, CO). Up to 30 ml of blood were collected using 18- to 21-gauge, 1.5- to 2-in needles (BD PrecisionGlide™, Becton Dickinson and Co., Franklin Lakes, NJ) attached to 6-ml syringes (Monoject™, Covidien™/Medtronic, Minneapolis, MN) and an extension set (Baxter International Inc., Deerfield, IL). The blood collection process lasted 2–4 min. Blood was immediately transferred into various tubes, including green top (lithium-heparin), royal blue top (sodium EDTA), red top with serum separator and red top tube (Becton Dickinson and Co.). Additionally, one drop (~1–5 μl) of whole blood from the lithium-heparin tube was used to determine glucose using a point-of-care glucometer (Accu-Chek®, Roche Diabetes Care, Inc., Indianapolis, IN). The blood tubes were insulated with bubble wrap in the field and transported on regular wet ice to Disney Animal Health’s in-house laboratory (Disney’s Animal Kingdom®, Orlando, FL), where the samples were processed within 12 h after blood collection.

Following venipuncture, morphometric measurements were taken including standard straight carapace length (SCL), minimum straight carapace length (SCL_min_), straight carapace width (SCW) and head width using Haglöf-Mantax (Haglöf Inc., Madison, MS) aluminium callipers. Minimum curved carapace length (CCL_min_) and curved carapace width (CCW) were measured using a flexible measuring tape. An external physical examination was also conducted (Deem & Harris, 2017; Page-Karjian & Perrault, 2021) to include evaluation of the eyes and nose and categorization of the scute and skin condition. Subjective body condition was assessed as being either ‘poor’, ‘fair’ or ‘good’, based on visual assessment, including evaluation of robust or sunken-appearing eyes, and subjective observation of soft tissue thickness of shoulder and neck. The presence and/or prevalence of epibionts with the potential to affect or indicate health, including cloacal marine leeches (*Ozobranchus* sp.), imbedded barnacles (*Platylepadidae* spp.) and commensal benthic taxa (e.g. invertebrates, macroalgae) were noted during the physical examination (Stacy & Innis, 2017, Stamper *et al*., 2005) and classified as ‘none’, ‘low’, ‘medium’ or ‘high’. Presence or absence of external lesions, including evidence of trauma (e.g. healed or active wounds) was recorded.

If previous tags were absent on the turtles, including scanning the front flippers for passive integrative transponder (PIT) tags with a microchip reader (Biomark GPR+, Boise, ID), Inconel tags (style 681; National Band and Tag Co., Newport, KY) were applied to the first scale proximal to the shell on the trailing edge of the turtles’ front flippers. In addition, a PIT tag was inserted into the triceps muscle of the right front flipper. Prior to tagging, the application site was cleaned using a sterile alcohol wipe at least three times.

Mass of sampled turtles was obtained during their return to sea after the nest camouflaging phase was complete. From 2016–17, soft nylon mesh (5.4-cm bar) scallop netting was placed on the sand ahead of the turtle’s projected route. The turtle was guided into the centre of the net, at which point she was quickly secured with the netting, which was then attached to a metal bar using a 10-cm shackle and lifted by two people. The shackle was fastened to a portable digital hanging scale (Handy Roughneck™, Unbranded, Northern Tool and Equipment, Burnsville, MN) to collect mass. From 2017–19, the metal bar was replaced with an aluminium tripod. For both methods, the turtle was elevated above the sand so that none of her extremities were touching the sand. After mass was recorded, the turtle was lowered and released. In 2019, the protocol was slightly adjusted to replace the scallop netting with a customized turtle sling. Scales were tared to the net or sling prior to weighing. BCI was calculated using the formula from Bjorndal *et al.* (2000):


$$ \mathrm{BCI}=\left(\frac{\mathrm{mass}}{{\mathrm{SCL}}^3}\right)\times \mathrm{10,000} $$

### Blood processing and analysis

At Disney’s in-house laboratory, well-mixed whole blood from each lithium-heparin tube was used to determine packed cell volume (PCV) using 75-mm non-heparinized capillary tubes (Jorgensen Laboratories, Inc. Loveland, CO) that were centrifuged for 3 min at 15 000 rpm (27 670 *g*) in a microhaematocrit centrifuge (Zip-IQ, LW Scientific, Lawrenceville, GA). After centrifugation, plasma colour and transparency were visually assessed for haemolysis, lipemia or other discoloration (Stacy & Innis, 2017). Plasma total protein (TP-R) and fibrinogen (heat precipitation method) were determined by standardized methods using a digital refractometer (AR200, Reichert® Technologies/Ametek®, Depew, NY) using the plasma portion from the capillary tube.

Up to four blood smears (from lithium-heparin tube) were prepared followed by air drying and fixation in >99% methanol (Thermo Fisher Scientific, West Palm Beach, FL). Slides were stained with Wright–Giemsa (SigmaAldrich Corp., St. Louis, MO) and evaluated by light microscopy for white blood cell (WBC) 200-cell differential of leukocytes (heterophils, lymphocytes, monocytes, eosinophils and basophils) and morphological assessment of red blood cells (RBCs), WBCs and thrombocytes. Number of immature RBCs per 100 mature RBCs was documented in addition to presence of RBC anisocytosis, polychromasia and basophilic stippling.

The remaining whole blood from all tubes was centrifuged (LW Ultra Select, LW Scientific, Lawrenceville, GA) at 3500 rpm (1507 *g*) for 15 min and plasma or serum was aliquoted into 2-ml cryogenic vials. All plasma and serum samples were stored frozen in an ultra-low cryogenic freezer at −80°C (Revco-EXF, Thermo Fisher Scientific, Marietta, OH) for up to 4 months prior to distribution and shipping in batches on dry ice. Analyses were run each year after completion of the nesting season at consistent diagnostic laboratories between 2017 and 2019.

Plasma samples (from lithium-heparin tubes) were submitted to the University of Miami’s Avian and Wildlife Laboratory (Miami, FL) for biochemical analyses, including alkaline phosphatase (ALP), amylase, aspartate aminotransferase (AST), blood urea nitrogen (BUN), creatine kinase (CK), calcium, phosphorus, chloride, cholesterol, gamma glutamyl-transferase (GGT), lipase, magnesium, potassium, sodium, total protein, triglycerides and uric acid using a Vitros® 250 dry chemistry analyser (Ortho-Clinical Diagnostics, Rochester, NY) and for plasma protein electrophoresis using a Helena SPIFE® 3000 (Helena Laboratories Corp., Beaumont, TX) electrophoresis system for albumin and total globulins. The calcium:phosphorus ratio (Ca:P) and albumin:globulin ratio were calculated.

Serum trace nutrient concentrations (from royal blue top tubes) including cobalt, copper, iron, manganese, molybdenum, selenium and zinc were measured using inductively coupled plasma-mass spectrometry (ICP-MS) using standards from Inorganic Ventures (Christiansburg, VA) at Michigan State University (MSU) Veterinary Diagnostic Laboratory (Lansing, MI). Serum iron concentrations were determined using an Olympus AU640e (Shinjuku City, Tokyo, Japan). β-hydroxybutyrate was quantified from lithium-heparinized plasma at MSU using the Catachem colorimetric assay (Catachem, Inc., Oxford, CT) on a Beckman Coulter AU series analyser (Brea, CA).

Serum vitamin concentrations (from royal blue top tubes) including retinol (i.e. vitamin A) and vitamin E isoforms (α-tocopherol, γ-tocopherol and δ-tocopherol) were determined using high-performance liquid chromatography (HPLC) at Eurofins Craft Technologies (Wilson, NC).

Fatty acid profiles were quantified in serum (from red top tubes) at Lipid Technologies (Austin, MN). Separation, detection and quantification were accomplished by capillary gas chromatography utilizing a 30-m Restek free fatty acid phase coating (Restek Corp., Bellefonte, PA) and EZChrom software (Scientific Software International Inc., Lincolnwood, IL).

### Statistical analysis

Measures of central tendency, range and 95% reference intervals with 90% confidence intervals for blood analytes are presented in standard international units for all study turtles (none had to be excluded due to injuries or abnormalities). Parametric methods for sample sizes ≥20 but <120 were used to calculate reference intervals, unless otherwise indicated (Friedrichs *et al.* 2012). Normality was assessed using the Shapiro–Wilk test, and outliers were detected using the Dixon–Reed test. All plasma samples included in the calculation of reference intervals had haemolysis and lipaemia scores ≤1+, which is considered to not cause interferences using dry chemistry analysis. Reference intervals could not be calculated for some variables due to low sample sizes (<20) or because most values fell below the detection limits (i.e. right-skewed data); these data are reported descriptively. Least-squares linear regressions (with data transformations as necessary) were used to compare mass and SCL; BCI and blood analytes; and total protein concentrations determined by refractometer and the Biuret method. Blood analyte data were combined by month across years and an analysis of variance (ANOVA) with Tukey’s *post hoc* tests was used to compare these data by month. Kruskal–Wallis tests with Dunn’s *post hoc* tests with Benjamini–Hochberg adjustment were used when the data did not meet the assumptions of normality. Kruskal–Wallis tests were also used to compare categorical data by month (e.g. subjective body condition, epibiont loads and presence of cloacal leeches).

## Results

### Physical examination and morphometrics

A total of 57 nesting loggerheads were sampled between 18 August 2016 and 17 August 2019. One turtle was sampled in 2016 (on 18 August), 9 in 2017 (from 4 May to 8 August), 24 in 2018 (from 12 May to 17 August) and 23 in 2019 (from 11 May to 17 August). Eight turtles were previously tagged, 48 turtles had no tags and new tags were applied and tagging information was missing for 1 turtle.

None of the nesting females showed evidence of behavioural or major external abnormalities or lesions; minor injuries were limited to the flippers and carapace. No turtles had external abnormalities to the rhamphothecae; however, 16 of 54 (30%) turtles presented with minimal, healed injuries including small divots in the carapacial scutes, minor and/or superficial damage to the posterior carapace, minor injuries to the hind limbs or healed partial amputations to the front flippers and hind limbs (Table 1). None of the turtles showed abnormal behaviour or abandoned their nesting attempts.

**Table 1:** Morphometrics and results of external physical exam from nesting loggerhead sea turtles (*Caretta caretta*) from the ACNWR, Florida, USA. For external physical exam, ‘poor’, ‘fair’ and ‘good’ correspond to subjective body condition and ‘none’, ‘low’, ‘medium’ and ‘high’ refer to prevalence of epibionts, *Stomatolepas* spp. barnacles and macroalgae

*Morphometrics*	Mean ± SD	Median	Range	*n*
Mass [kg]	98.7 ± 18.3	97.2	64.6–139.6	41
Body condition index	1.35 ± 0.12	1.33	1.14–1.66	40
Straight carapace length [cm]	89.9 ± 5.4	89.8	80.7–102.5	53
Minimum straight carapace length [cm]	88.0 ± 4.9	87.6	79.5–96.5	50
Straight carapace width [cm]	67.7 ± 5.5	67.3	57.4–87.0	50
Minimum curved carapace length [cm]	95.3 ± 6.0	95.3	80.1–109.8	52
Curved carapace width [cm]	88.0 ± 5.8	88.1	77.4–101.2	51
Head width [cm]	19.1 ± 2.2	18.5	15.5–24.1	48
*External physical exam*	None	Poor/low	Fair/moderate	Good/high
Subjective body condition		0/56 (0%)	12/56 (21%)	44/56 (79%)
Epibiont load	0/57 (0%)	30/57 (53%)	24/57 (42%)	3/57 (5%)
*Stomatolepas* spp. barnacles	7/54 (13%)	40/54 (74%)	5/54 (9%)	2/54 (4%)
Macroalgae	7/53 (13%)	24/53 (45%)	14/53 (26%)	8/53 (15%)
*Injuries/leeches*	Yes		No	
Cloacal leeches present	37/44 (84%)		7/44 (16%)	
Rhamphotheca injury	0/56 (0%)		56/56 (100%)	
Flipper/carapace injury	16/54 (30%)		38/54 (70%)	

**Figure 2:**
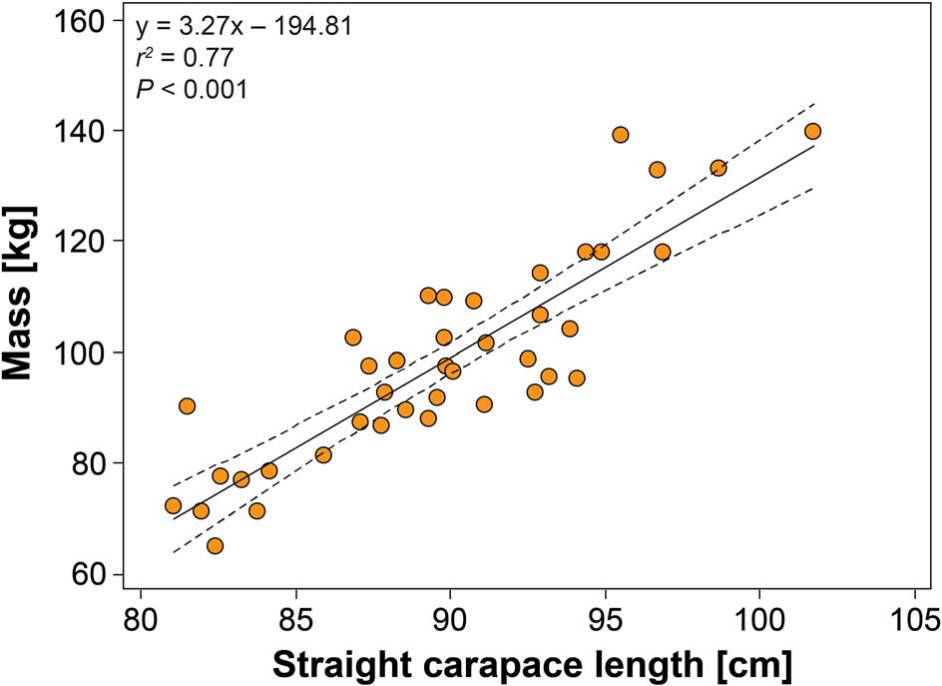
Relationship between mass (kg) and standard straight carapace length (cm) for nesting loggerhead sea turtles (*Caretta caretta*) from the ACNWR. The trend was significant and the equation for the line-of-best-fit is shown with the 95% confidence interval of the regression line.

All turtles were in fair to good body condition (Table 1) based on subjective observation of thickness of shoulder and neck soft tissues and BCI (Harris *et al.* 2017). Measures of central tendency and range for mass, BCI and morphometrics are reported in Table 1. Mass and SCL showed a strong linear relationship (*r^2^* = 0.77; *P* < 0.001; *n* = 40; Fig. 2).

Generally, turtles included in this study had low to moderate skin and carapace epibiota that consisted largely of *Stomatolepas* spp. barnacles and macroalgae. The majority of turtles (84%: 37 of 44) had leeches within their cloaca. Subjective BCI and prevalence of cloacal leeches, *Stomatolepas* spp. barnacles and macroalgae did not significantly differ by month (*P* > 0.050 in all cases). Epibiota load (scored as none, low, medium and high) significantly differed by month (*H*(3) = 13.148; *P* = 0.004), with loggerheads nesting in May having significantly fewer epibionts than loggerheads nesting in July (*P* = 0.002) and August (*P* = 0.016).

### Reference intervals

Samples with moderate (2+) to marked (3+) haemolysis (*n* = 12) and moderate (2+) lipaemia (*n* = 1) were removed from calculation of reference intervals (Stacy & Innis 2017; Stacy *et al.,* 2019). Lymph contamination was not observed in any blood samples during collection. Measures of central tendency, range and reference intervals in Standard International units are reported for haematology and plasma biochemistry data in Table 2; protein electrophoresis, vitamins, trace nutrients and BHB in Table 3; and fatty acids in Table 4. Supplemental Tables 1 and 2 report the same blood analyte data in conventional units. Light microscopic evaluation of blood cells indicated that polychromasia, anisocytosis, haemoparasites, heterophil toxic change or other potentially clinically relevant findings were absent in all samples. Thrombocytes were adequate in all samples. For three turtles, the PCV was < 20% *(n =* 3; 15–19% during July and August). A very strong positive relationship existed between estimated total protein (in g/l) as determined by the refractometer (TP-R) and total protein (in g/l) as determined by the Biuret method (TP-B; *r^2^* = 0.84; *P* < 0.001; *n* = 30). This relationship is described by the formula:


\begin{align*} \mathrm{Total}\ \mathrm{protein}\ \left(\mathrm{TP}-\mathrm{R}\right)&=\left[1.254\times \mathrm{total}\ \mathrm{protein}\ \left(\mathrm{TP}-\mathrm{B}\right)\right] - 5.124 \end{align*}

**Table 2:** Measures of central tendency, range and reference intervals (with 90% confidence intervals for upper and lower limits) for haematologic and plasma biochemical data in Standard International units for nesting loggerhead sea turtles (*Caretta caretta*) from the ACNWR. Parametric methods for sample sizes ≥20 but <120 were used to calculate reference intervals, unless otherwise indicated by footnotes. Normality was assessed using the Shapiro–Wilk test, whilst outliers were detected using the Dixon–Reed test. All plasma samples had haemolysis and lipaemia scores ≤1+, which is not considered to cause interference using dry chemistry analysis. Reference intervals could not be calculated for some variables due low sample sizes (<20) or because the majority of the values fell below the detection limit (i.e. right-skewed data)

Analyte	Mean ± SD	Median	Range	*n*	RI	LRL 90% CI	URL 90% CI	Data distribution, RI method, transformation
*Haematology*								
Packed cell volume [L/L]	0.27 ± 0.06	0.28	0.15–0.38	25	0.15–0.39	0.11–0.18	0.35–0.42	G, P, N
Immature RBC/100 mature RBC	0	0	0	38				
White blood cells [×10^9^/L]	6.67 ± 2.15	6.10	3.90–12.70	38	3.57–11.39	3.11–4.09	9.92–13.07	G, P, L
Heterophils [×10^9^/L]	3.15 ± 1.25	2.90	1.10–6.20	33	1.34–6.34	1.10–1.64	5.20–7.73	G, P, L
Immature heterophils [×10^9^/L]	0.01 ± 0.02	0	0–0.07	33				
Total heterophils [×10^9^/L]	3.16 ± 1.25	2.90	1.10–6.20	33	1.35–6.38	1.10–1.64	5.23–7.78	G, P, L
Lymphocytes [×10^9^/L]	2.53 ± 0.86	2.40	1.20–5.50^a^	33	1.10–3.77	0.75–1.44	3.43–4.12	G, P, N
Monocytes [×10^9^/L]	0.47 ± 0.23	0.43	0.12–1.30	33	0.17–1.05	0.14–0.22	0.84–1.32	G, P, L
Eosinophils [×10^9^/L]	0.69 ± 0.60	0.50	0.09–2.30	33	0.08–2.73	0.05–0.13	1.75–4.26	G, P, L
Basophils [×10^9^/L]	0.03 ± 0.06	0	0–0.19	33				
Heterophil:lymphocyte ratio	1.36 ± 0.80	1.15	0.41–5.00^b^	33	0.34–2.16	0.11–0.58	1.92–2.39	G, P, N
*Biochemistry*								
Alkaline phosphatase [μkat/L]		<0.17	<0.17–0.67^c^	43				
Amylase [μkat/L]	8.78 ± 3.27	8.73	2.27–18.69	43	2.36–15.21	0.91–3.78	13.76–16.63	G, P, N
Aspartate aminotransferase [μkat/L]	3.29 ± 1.51	2.92	1.86–9.63	43	1.94–7.45	1.83–2.08	5.25–15.67	G, P, B
Blood urea nitrogen [mmol/L]	4.3 ± 1.9	3.9	1.1–10.0	43	1.7–8.7	1.4–2.1	7.4–10.2	G, P, B
Creatine phosphokinase [μkat/L]	13.90 ± 12.80	11.55	3.82–86.38^d^	43	3.90–29.55	3.10–4.90	23.50–37.15	G, P, L
Calcium [mmol/L]	2.8 ± 1.1	2.7	1.2–7.9	41	1.3–4.8	1.1–1.7	3.8–5.8	NG, R, L
Phosphorus [mmol/L]	2.9 ± 0.7	2.8	1.7–5.6^c^	43	1.8–3.8	1.6–2.0	3.6–4.0	G, P, N
Calcium:phosphorus ratio	0.96 ± 0.23	0.96	0.45–1.60	41	0.52–1.41	0.41–0.62	1.31–1.52	G, P, N
Chloride [mmol/L]	118 ± 6	117	106–134	43	106–131	103–109	128–133	G, P, N
Cholesterol [mmol/L]	4.9 ± 1.6	4.6	2.5–8.3	43	2.5–8.6	2.2–2.9	7.5–9.9	G, P, L
Gamma glutamyl-transferase [μkat/L]		<0.08	<0.08–0.17	43				

(Continued)

**Table 2:** Continued

Analyte	Mean ± SD	Median	Range	*n*	RI	LRL 90% CI	URL 90% CI	Data distribution, RI method, transformation
Glucose DCA [mmol/L]	4.4 ± 0.9	4.4	2.2–6.4	43	2.7–6.1	2.3–3.1	5.7–6.5	G, P, N
Glucose glucometer [mmol/L]	4.3 ± 0.7	4.4	3.0–5.2	12				
Lipase [μkat/L]		0.10	<0.10–0.93	43	<0.10–0.98	<0.10	0.68–1.48	NG, R, B
Magnesium [mmol/L]	2.4 ± 0.4	2.4	1.8–3.4	43	1.7–3.1	1.5–1.8	2.9–3.2	G, P, N
Potassium [mmol/L]	3.8 ± 0.5	3.6	3.0–5.2	43	2.9–4.8	2.7–3.1	4.5–5.1	G, P, L
Sodium [mmol/L]	144 ± 4	144	133–152	43	135–152	133–137	150–154	G, P, N
Triglycerides [mmol/L]	6.2 ± 5.3	5.8	0.7–18.6	43	0.4–41.3	0.3–0.7	26.5–55.0	NG, R, L
Uric acid [mmol/L]		0.04	<0.006–0.059	43	0.01–0.06	<0.006–0.01	0.05–0.06	G, P, N

Abbreviations: B, Box–Cox transformation; CI, confidence interval; DCA, dry chemistry analyzer; G, Gaussian distribution; L, logarithmic transformation; LRL, lower reference limit; N, no transformation; NG, non-Gaussian distribution; P, parametric method; R, robust method; RI, reference interval; SD, standard deviation; URL, upper reference limit.

a ^a^5.50 × 10^9^ cells/L was an outlier and was removed from reference interval calculations. The second highest value was 3.70 × 10^9^ cells/L.

b ^b^5.00 was an outlier and was removed from reference interval calculations. The second highest value was 2.32.

c ^c^0.67 μkat/L was an outlier. The second highest value was 0.47 μkat/L.

d ^d^86.38 μkat/L was an outlier and was removed from reference interval calculations. The second highest value was 26.08 μkat/L.

**Table 3:** Measures of central tendency, range and reference intervals (with 90% confidence intervals for upper and lower limits) for plasma proteins, trace nutrients, vitamins and β-hydroxybutyrate in Standard International units for nesting loggerhead sea turtles (*Caretta caretta*) from the ACNWR. Parametric methods for sample sizes ≥20 but <120 were used to calculate reference intervals, unless otherwise indicated by footnotes. Normality was assessed using the Shapiro–Wilk test, whilst outliers were detected using the Dixon–Reed test. All plasma samples had haemolysis and lipaemia scores ≤1+, which is not considered to cause interference using dry chemistry analysis. Reference intervals could not be calculated for some variables due low sample sizes (<20) or because the majority of the values fell below the detection limit (i.e. right-skewed data)

Analyte	Mean ± SD	Median	Range	*n*	RI	LRL 90% CI	URL 90% CI	Data distribution, RI method, transformation
*Plasma proteins*								
Total protein-Biuret [g/l]	44 ± 10	43	29–71	39	24–64	20–29	59–68	G, P, N
Total protein-Refractometer [g/l]	49 ± 16	46	29–93	25	26–86	22–31	72–103	G, P, L
Albumin [g/l]	9.6 ± 2.2	9.3	6.3–14.5	39	5.4–13.8	4.4–6.4	12.8–14.8	G, P, N
Globulins [g/l]	33.0 ± 8.6	32.3	20.8–56.9	39	16.2–49.9	12.2–20.1	45.9–53.8	G, P, N
Albumin:globulin	0.34 ± 0.07	0.34	0.22–0.49	39	0.21–0.48	0.18–0.24	0.45–0.51	G, P, N
Fibrinogen [g/l]	1.4 ± 1.8	1.0	0–5.0	18				
*Trace nutrients*								
Cobalt [nmol/l]	32.2 ± 58.7	11.5	2.7–287.6	37	1.2–116.6	0.7–2.2	58.9–218.9	NG, R, L
Copper [μmol/l]	6.8 ± 2.0	6.0	4.6–13.2	37	1.4–10.5	0.2–3.0	9.0–12.0	NG, R, N
Iron [μmol/l]	9.0 ± 5.0	7.7	2.9–26.0	37	2.9–21.8	2.2–3.6	17.0–27.9	G, P, L
Manganese [nmol/l]	360.4 ± 349.5	273.0	103.8–1980.4^a^	37	83.7–833.7	61.9–111.0	628.0–1104.9	G, P, L
Molybdenum [nmol/l]	537.3 ± 393.4	422.2	115.1–2322.0^b^	37	163.1–1141.8	134.3–211.1	901.9–1439.3	G, P, L
Selenium [nmol/l]	4.2 ± 2.9	3.1	1.3–14.9	37	1.1–11.2	0.8–1.5	8.5–14.8	G, P, L
Zinc [μmol/l]	17.9 ± 7.2	16.1	11.0–52.8^c^	37	10.7–25.5	9.6–11.9	22.9–28.5Do	G, P, L
*Vitamins*								
α-tocopherol [μmol/l]	37.3 ± 102.4	6.7	0.4–580.0	34	0.2–264.9	0.1–0.5	106.1–661.5	G, P, L
γ-tocopherol [μmol/l]		<0.1	<0.1–4.2^d^	34				
δ-tocopherol [μmol/l]		<0.1	<0.1–0.7	34				
Retinol [μmol/l]	2.22 ± 2.73	1.20	0.60–10.96	34	0.24–5.95	0.16–0.43	3.32–9.64	NG, R, L
*β-hydroxybutyrate*								
β-hydroxybutyrate [mmol/l]	1.36 ± 0.77	1.27	0.30–3.86	37	0.41–3.39	0.32–0.53	2.63–4.36	G, P, L

a ^a^1980.4 nmol/l was an outlier and was removed from reference interval calculations. The second highest value was 1015.7 nmol/l.

b ^b^2322.0 nmol/l was an outlier and was removed from reference interval calculations. The second highest value was 1295.1 nmol/l.

c ^c^52.8 μmol/l was an outlier and was removed from reference interval calculations. The second highest value was 31.8 μmol/l.

d ^d^4.2 μmol/l was an outlier and was removed from reference interval calculations. The second highest value was 1.0 μmol/l.

**Table 4:** Measures of central tendency, range and reference intervals (with 90% confidence intervals for upper and lower limits) for fatty acids (in μg/ml) in Standard International units for nesting loggerhead sea turtles (*Caretta caretta*) from the ACNWR. Parametric methods for sample sizes ≥20 but <120 were used to calculate reference intervals, unless otherwise indicated by footnotes. Normality was assessed using the Shapiro–Wilk test, whilst outliers were detected using the Dixon–Reed test. All plasma samples had haemolysis and lipaemia scores ≤1+, which is not considered to cause interference using dry chemistry analysis. Reference intervals could not be calculated for some variables because the majority of the values fell below the detection limit (i.e. right-skewed data)

Lipid number	Fatty acid	Mean ± SD	Median	Range	*n*	RI	LRL 90% CI	URL 90% CI	Data distribution, RI method, transformation
12:0	Lauric acid	0.9 ± 1.9	0	0–9.4^a^	37				
14:0	Myristic acid	385.4 ± 259.2	362.9	61.3–953.4	37	53.7–1687.0	37.4–94.0	1272.1–2477.5	NG, R, L
14:1	Myristoleic acid	46.3 ± 27.5	45.3	4.4–111.4	37	4.7–113.1	1.2–10.5	93.0–135.0	G, P, B
15:0	Pentadecanoic acid	22.8 ± 16.4	19.3	0.9–57.8	37	1.1–67.2	0.1–3.1	52.4–84.2	G, P, B
15:1	Pentadecenoic acid	2.1 ± 1.4	2.2	0–4.5	37	0.001–7.1	0–0.3	5.9–8.3	NG, R, B
16:0	Palmitic acid	1054.9 ± 604.0	1086.9	205.8–2611.0	37	166.0–2582.1	81.8–287.0	2099.8–3124.1	G, P, B
16:1ω5	Palmitovaccenic acid	0	0	0	37				
16:1ω7	Palmitoleic acid	638.8 ± 454.0	608.9	104.8–1925.0	37	100.0–2334.1	68.4–146.2	1596.5–3412.5	G, P, L
17:1	Heptadecenoic acid	45.4 ± 43.7	31.1	1.2–224.8^b^	37	0.5–131.8	0–3.2	99.8–169.0	G, P, B
18:0	Stearic acid	345.3 ± 170.1	335.2	89.4–810.3	37	106.7–864.3	82.9–137.3	671.6–1112.2	G, P, L
18:1ω9	Oleic acid	2017.3 ± 1254.8	1981.2	373.3–5317.3	37	437.5–6195.2	317.8–602.2	4500.4–8528.3	G, P, L
18:1ω7	Vaccenic acid	0	0	0	37				
18:2ω6	Linoleic acid	98.1 ± 67.0	76.0	0.7–295.8	37	4.6–260.8	0.1–15.3	210.6–315.9	G, P, B
18:3ω6	Linolelaidic acid	5.4 ± 6.2	2.5	0–20.5	37	0–30.3	0–0.02	19.1–45.5	G, P, B
18:3ω3	α-Linolenic acid	11.4 ± 13.7	5.4	0–55.9	37	0–54.2	0–0.2	36.0–78.0	G, P, B
18:4ω3	Stearidonic acid	0	0	0	37				
20:0	Arachidic acid	4.3 ± 4.4	3.4	0–19.0	37	0–24.2	0–0.1	16.6–30.3	NG, R, B
20:1ω7	Paullinic acid	37.3 ± 27.2	37.6	3.4–95.8	37	0.5–124.1	0–4.0	101.2–145.9	NG, R, L
20:1ω9	Gondoic acid	13.4 ± 18.0	7.7	0–78.6	37	0–71.2	0–0.1	46.2–102.4	NG, R, B
20:2ω6	Dihomo-linoleic acid	7.7 ± 5.9	6.5	0.9–32.8^c^	37	1.6–20.8	1.2–2.2	15.3–28.5	G, P, L
20:3ω9	Mead acid	4.0 ± 5.2	2.3	0–24.1	37	0–19.2	0–0.1	12.0–27.6	NG, R, B

(Continued)

**Table 4:** Continued

Lipid number	Fatty acid	Mean ± SD	Median	Range	*n*	RI	LRL 90% CI	URL 90% CI	Data distribution, RI method, transformation
20:3ω6	Dihomo-γ-linolenic acid	6.5 ± 4.5	5.2	0–14.9	37	0.04–22.3	0–0.6	18.1–27.7	NG, R, B
20:4ω6	Arachidonic acid	309.2 ± 152.3	282.8	103.9–681.3	37	72.8–727.1	46.7–105.3	628.1–893.7	NG, R, B
20:3ω3	Dihomo-α-linolenic acid	1.2 ± 1.4	0	0–5.2	37				
20:4ω3	Eicosatetraenoic acid	2.0 ± 3.2	0.8	0–14.5^d^	37				
20:5ω3	Eicosapentaeonic acid	63.5 ± 88.3	30.7	2.8–428.6^e^	37	3.2–267.6	1.9–5.5	155.8–459.7	G, P, L
22:0	Behenic acid	36.9 ± 18.9	34.8	6.4–81.8	37	9.6–104.1	7.2–12.8	78.0–138.8	G, P, L
22:1ω9	Erucic acid	1.5 ± 1.8	1.0	0–6.1	37				
22:4ω6	Docosatetraenoic acid	30.4 ± 17.7	26.8	2.2–62.1	37	2.6–71.8	0.2–6.7	59.9–84.6	G, P, B
22:5ω6	Osbond acid	7.9 ± 4.2	7.0	0.6–18.3	37	1.0–17.3	0.2–2.1	14.7–20.1	G, P, B
22:5ω3	Clupanodonic acid	62.5 ± 35.9	61.2	14.5–159.8	37	11.4–155.6	6.6–18.1	125.0–190.8	G, P, B
24:0	Lignoceric acid	5.0 ± 8.7	1.0	0–30.5	37	0–74.6	0	40.0–113.7	NG, R, B
22:6ω3	Cervonic acid	109.6 ± 80.2	87.1	25.5–364.4	37	22.2–337.9	16.0–30.9	243.4–469.1	G, P, L
24:1	Nervonic acid	10.0 ± 12.2	6.9	0.6–65.8^f^	37	0.8–40.0	0.5–1.3	24.8–64.6	G, P, L
	Other	195.2 ± 130.1	167.2	33.4–671.5	37	43.3–586.6	31.6–59.3	428.4–803.3	G, P, L
	Sum	5581.7 ± 3237.1	5566.3	1103.9–14 043.8	37	1291.3–16 626.0	948.9–1757.4	12 217.1–22 625.9	G, P, L
	Saturates	1855.3 ± 1047.8	1907.0	367.6–4374.6	37	311.1–4511.0	161.4–522.9	3670.1–5458.0	G, P, B
	Monoenes	2812.1 ± 1781.1	2722.6	499.9–7559.6	37	577.7–8906.0	415.4–803.5	6403.7–12 386.1	G, P, L
	PUFA	719.2 ± 356.1	690.9	203.1–1667.2	37	222.2–1800.0	172.7–286.0	1398.7–2316.5	G, P, L
	HUFA	613.4 ± 300.6	596.3	179.0–1411.7	37	193.6–1514.2	151.0–248.1	1181.6–1940.5	G, P, L

(Continued)

**Table 4:** Continued

Lipid number	Fatty acid	Mean ± SD	Median	Range	*n*	RI	LRL 90% CI	URL 90% CI	Data distribution, RI method, transformation
	Total ω3	250.1 ± 192.1	201.6	54.3–971.0^g^	37	57.5–635.4	42.9–77.1	473.7–852.3	G, P, L
	Total ω6	465.1 ± 225.1	469.9	147.8–999.9	37	121.3–1022.4	80.2–174.0	848.8–1217.5	G, P, B
	Total ω9	2088.5 ± 1289.2	2047.2	383.3–5446.9	37	458.8–6369.0	334.1–630.1	4637.8–8746.5	G, P, L
	ω6/ω3	2.30 ± 0.95	2.40	0.50–3.90	37	0.36–4.11	0–0.87	3.68–4.52	G, P, B
	AA:EPA	13.76 ± 11.70	8.80	0.70–39.40	37	0.53–49.48	0.14–1.38	35.80–66.57	G, P, B

Abbreviations: ω3, omega-3 fatty acids; ω6, omega-6 fatty acids; ω9, omega-9 fatty acids; AA:EPA, arachidonic acid to eicosapentaenoic acid ratio; HUFA, highly unsaturated fatty acid; PUFA, polyunsaturated fatty acid;

a ^a^9.4 μg/ml was an outlier. The second highest value was 4.5 μg/ml.

b ^b^224.8 μg/ml was an outlier and was removed from reference interval calculations. The second highest value was 111.4 μg/ml.

c ^c^32.8 μg/ml was an outlier and was removed from reference interval calculations. The second highest value was 20.0 μg/ml.

d ^d^14.5 μg/ml was an outlier and was removed from reference interval calculations. The second highest value was 8.6 μg/ml.

e ^e^428.6 μg/ml was an outlier and was removed from reference interval calculations. The second highest value was 245.0 μg/ml.

f ^f^65.8 μg/ml was an outlier and was removed from reference interval calculations. The second highest value was 36.3 μg/ml.

g ^g^971.0 μg/ml was an outlier and was removed from reference interval calculations. The second highest value was 647.9 μg/ml.

### Correlations of BCI with blood analytes

**Figure 3:**
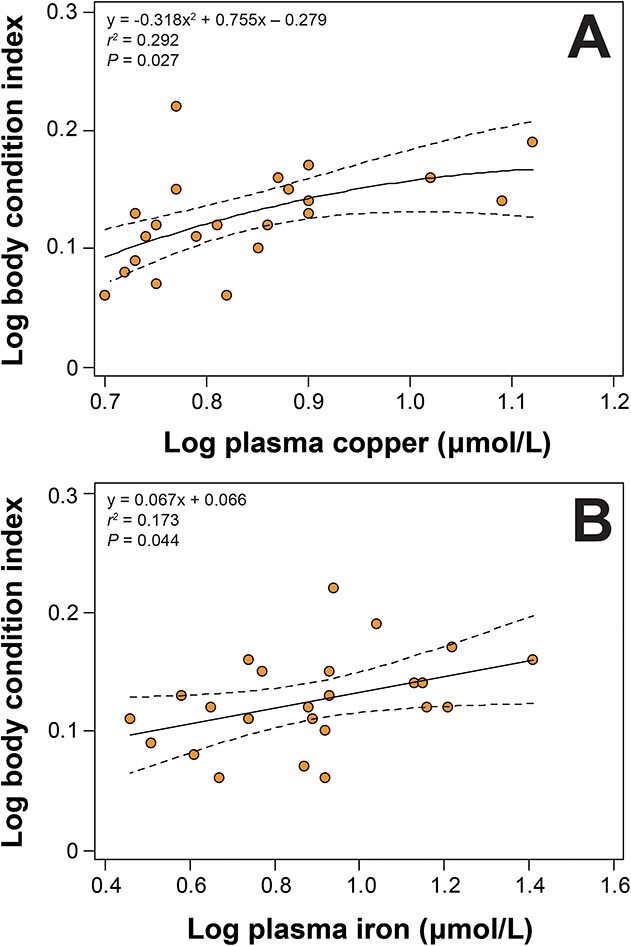
Relationship between BCI and plasma (a) copper and (b) iron in nesting loggerhead sea turtles (*Caretta caretta*) from the ACNWR. Data were log-transformed. The trends were significant and the equations for the line-of-best-fit are shown with the 95% confidence intervals of the regression line.

A significant polynomial relationship existed between BCI and copper (y = −0.318x^2^ + 0.755x −0.168; *r^2^* = 0.292; *P* = 0.027; Fig. 3a), whilst a linear relationship existed between BCI and iron (y = 0.067x + 0.066; *r^2^* = 0.173; *P* = 0.044; Fig. 3b).

### Correlations of morphometric data and blood analytes with month of nesting season

**Figure 4:**
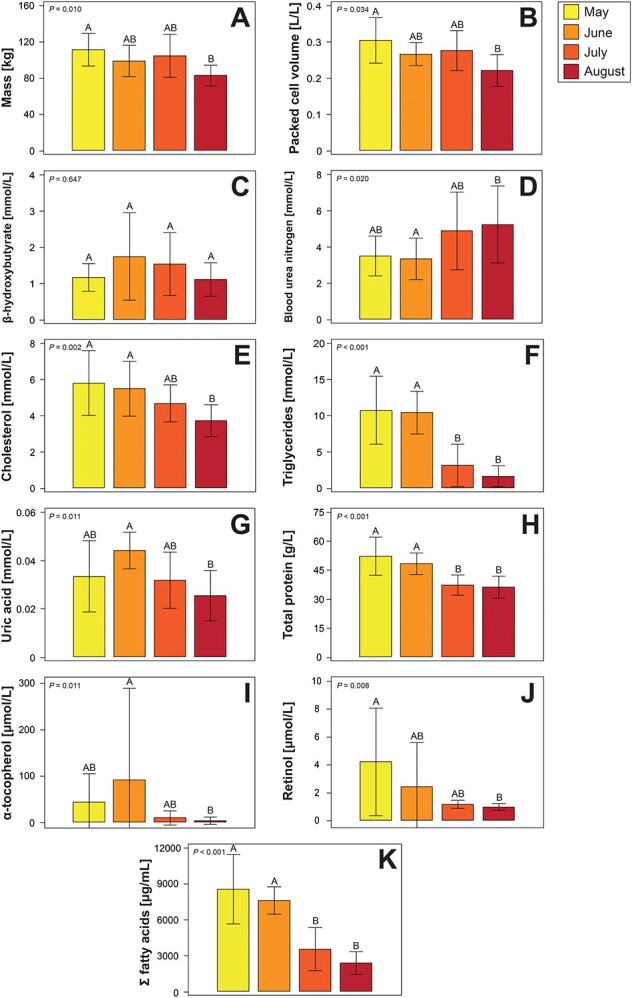
Mean ± standard deviation of mass and select blood analytes showing significant trends (with the exception of β-hydroxybutyrate, which was not significant, but tended to increase mid-season) by month of nesting season in nesting loggerhead sea turtles (*Caretta caretta*) from the ACNWR. Morphometric results include (A) mass, whilst included blood analytes are (B) PCV, (C) β-hydroxybutyrate, (D) blood urea nitrogen, (E) cholesterol, (F) triglycerides, (G) uric acid, (H) total protein, (I) α-tocopherol, (J) retinol and (K) sum of all detected fatty acids. ANOVA with Tukey’s *post hoc* tests were used to compare data by month, with Kruskal–Wallis tests with Dunn’s *post hoc* tests with Benjamini–Hochberg adjustment were used when the data did not meet the assumptions of the ANOVA. Different letters above each column represent significant differences by month at *P* < 0.050.

**Figure 5:**
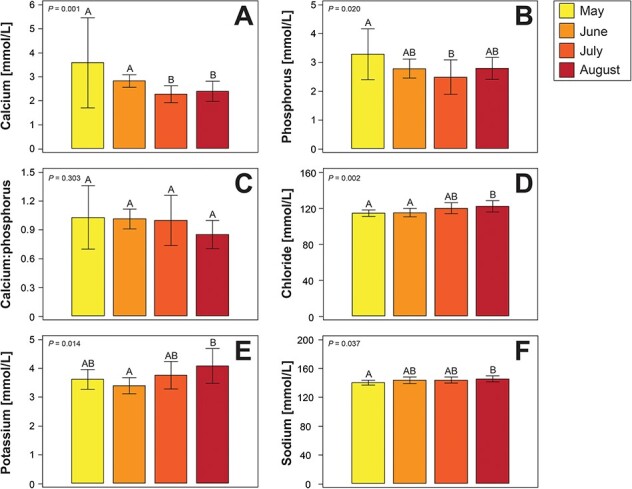
Mean ± standard deviation of electrolytes and minerals showing significant trends (with the exception of calcium:phosphorus, which was not significant) by month of nesting season in nesting loggerhead sea turtles (*Caretta caretta*) from the ACNWR. Electrolytes and minerals include (A) calcium, (B) phosphorus, (C) calcium:phosphorus, (D) chloride, (E) potassium and (F) sodium. ANOVA with Tukey’s *post hoc* tests were used to compare data by month, with Kruskal–Wallis tests with Dunn’s *post hoc* tests with Benjamini–Hochberg adjustment were used when the data did not meet the assumptions of the ANOVA. Different letters above each column represent significant differences by month at *P* < 0.050.

Complete statistical results of ANOVA and Kruskal–Wallis tests comparing morphometric data and blood analytes by month are shown in Tables 5 and 6, with graphical representation of select analytes provided in Figs. 4 and 5. Mass was the only morphometric analyte showing significant differences by month of nesting season, with an overall decline across nesting season. Blood analytes that decreased by month included PCV, amylase, calcium, cholesterol, lipase, phosphorus, triglycerides, uric acid, total protein by Biuret and refractometer, albumin, total globulins, iron, zinc, α-tocopherol, retinol and 23 fatty acids. Blood analytes that increased included BUN, chloride, potassium and sodium. Whilst not significant, β-hydroxybutyrate concentrations by month are shown in Fig. 4c.

**Table 5:** Statistical results of morphometric data and blood analytes by month of nesting season in nesting loggerhead sea turtles (*Caretta caretta*) from the ACNWR. Sample sizes are provided parenthetically. ANOVA with Tukey’s *post hoc* tests were used to compare data by month, with Kruskal–Wallis (KW) tests with Dunn’s *post hoc* tests with Benjamini–Hochberg adjustment were used when the data did not meet the assumptions of the ANOVA. The mean values are shown by month with different superscript letters indicating significant differences (at *P* < 0.050) as determined by *post hoc* tests. The test statistics are provided (F for ANOVA, H for KW), in addition to the degrees of freedom (df), the *P-*value and if transformations were performed

**Analyte**	**May**	**June**	**July**	**August**	**Test**	**F or H**	**df**	***P***	**Transformation**
*Morphometrics*									
Mass [kg]	108.7^A^ (8)	97.7^AB^ (7)	103.4^AB^ (7)	83.0^B^ (9)	ANOVA	4.402	3, 37	0.010	None
*Haematology*									
Packed cell volume [l/l]	0.30^A^ (9)	0.27^AB^ (3)	0.28^AB^ (5)	0.22^B^ (8)	ANOVA	3.490	3, 21	0.034	None
*Plasma biochemistry*									
Amylase [μkat/l]	11.07^A^ (12)	10.92^A^ (8)	6.81^B^ (10)	6.87^B^ (13)	ANOVA	9.337	3, 39	<0.001	Logarithmic
Blood urea nitrogen [mmol/l]	3.5^AB^ (12)	3.5^A^ (8)	4.9^AB^ (10)	5.3^B^ (13)	ANOVA	3.657	3, 39	0.020	Logarithmic
Calcium [mmol/l]	3.6^A^ (12)	2.8^A^ (8)	2.3^B^ (10)	2.4^B^ (13)	KW	15.759	3	0.001	None
Phosphorus [mmol/l]	3.3^A^ (12)	2.8^AB^ (8)	2.5^B^ (10)	2.8^AB^ (13)	ANOVA	3.684	3, 39	0.020	Logarithmic
Chloride [mmol/l]	114^A^ (12)	115^A^ (8)	120^AB^ (10)	122^B^ (13)	ANOVA	5.812	3, 39	0.002	None
Cholesterol [mmol/l]	5.8^A^ (12)	5.5^A^ (8)	4.7^AB^ (10)	3.7^B^ (13)	ANOVA	5.875	3, 39	0.002	Logarithmic
Lipase [μkat/l]	0.3^A^ (12)	0.2^AB^ (8)	0.1^B^ (10)	0.1^B^ (13)	KW	14.684	3	0.002	None
Potassium [mmol/l]	3.7^AB^ (12)	3.4^A^ (8)	3.8^AB^ (10)	4.1^B^ (13)	ANOVA	4.011	3, 39	0.014	None
Sodium [mmol/l]	141^A^ (12)	144^AB^ (8)	144^AB^ (10)	146^B^ (13)	ANOVA	3.126	3, 39	0.037	None
Triglycerides [mmol/l]	10.8^A^ (12)	10.4^A^ (8)	3.1^B^ (10)	1.7^B^ (13)	ANOVA	34.558	3, 39	<0.001	Logarithmic
Uric acid [mmol/l]	0.033^AB^ (12)	0.044^A^ (8)	0.032^AB^ (10)	0.026^B^ (13)	ANOVA	4.250	3, 39	0.011	None
*Protein electrophoresis*									
Total protein Biuret [g/l]	52^A^ (12)	48^A^ (8)	37^B^ (10)	36^B^ (9)	ANOVA	13.106	3, 35	<0.001	Logarithmic
Total protein refractometer [g/l]	65^A^ (9)	52^AB^ (3)	40^B^ (5)	37^B^ (8)	ANOVA	12.278	3, 21	<0.001	Logarithmic
Albumin [g/l]	11.0^A^ (12)	10.6^AC^ (8)	8.1^B^ (10)	8.5^BC^ (9)	ANOVA	7.000	3, 35	0.001	None
Total globulins [g/l]	39.8^A^ (12)	36.5^A^ (8)	27.9^B^ (10)	26.6^B^ (9)	ANOVA	10.272	3, 35	<0.001	Logarithmic
*Trace nutrients*									
Iron [μmol/l]	12.0^A^ (12)	11.8^AC^ (8)	6.8^BC^ (8)	4.5^B^ (9)	ANOVA	15.186	3, 33	<0.001	Logarithmic
Zinc [μmol/l]	26.1^A^ (12)	16.6^AB^ (8)	9.9^B^ (8)	19.7^AB^ (9)	KW	11.326	3	0.010	None
*Vitamins*									
α-tocopherol [μmol/l]	44.6^AB^ (9)	91.8^A^ (8)	10.8^AB^ (9)	4.5^B^ (8)	ANOVA	4.377	3, 30	0.011	Logarithmic
Retinol [μmol/l]	4.23^A^ (9)	2.44^AB^ (8)	1.18^AB^ (9)	1.00^B^ (8)	KW	11.860	3	0.008	None

**Table 6:** Statistical results of fatty acids by month of nesting season in nesting loggerhead sea turtles (*Caretta caretta*) from the ACNWR. Samples sizes are provided parenthetically by the name of the month. ANOVA with Tukey’s *post hoc* tests were used to compare data by month, with KW tests with Dunn’s *post hoc* tests with Benjamini–Hochberg adjustment were used when the data did not meet the assumptions of the ANOVA. The mean values (in μg/ml) are shown by month with different superscript letters indicating significant differences (at *P* < 0.050) as determined by *post hoc* tests. The test statistics are provided (F for ANOVA, H for KW), in addition to the degrees of freedom (df), the *P-*value and if transformations were performed

**Lipid number**	**Fatty acid**	**May (*n* = 12)**	**June (*n* = 8)**	**July (*n* = 10)**	**August (*n* = 9)**	**Test**	**F or H**	**df**	***P***	**Transformation**
12:0	Lauric acid	1.7^AB^	1.8^A^	0.16^AB^	0^B^	KW	10.711	3	0.013	None
14:0	Myristic acid	661.4^A^	534.8^AB^	230.2^B^	133.8^B^	KW	24.097	3	<0.001	None
14:1	Myristoleic acid	78.0^A^	51.2^AB^	29.0^B^	31.2^B^	KW	19.110	3	<0.001	None
15:0	Pentadecanoic acid	27.7^AB^	43.8^A^	12.4^BC^	7.6^C^	KW	25.561	3	<0.001	None
16:0	Palmitic acid	1585.9^A^	1503.7^A^	688.2^B^	410.4^B^	KW	26.213	3	<0.001	None
16:1ω7	Palmitoleic acid	1118.9^A^	815.1^AB^	359.7^BC^	219.4^C^	KW	26.850	3	<0.001	None
17:1	Heptadecenoic acid	67.4^A^	68.7^A^	20.6^B^	24.4^AB^	KW	12.155	3	0.007	None
18:0	Stearic acid	476.9^A^	466.8^A^	236.3^B^	174.2^B^	KW	20.455	3	<0.001	None
18:1ω9	Oleic acid	3167.9^A^	2716.3^A^	1293.3^B^	850.1^B^	KW	25.859	3	<0.001	None
18:2ω6	Linoleic acid	116.4^AB^	148.5^A^	56.7^B^	56.4^B^	KW	16.842	3	<0.001	None
20:0	Arachidic acid^a^	6.1^A^	6.9^A^	3.2^AB^	0.9^B^	KW	20.934	3	<0.001	None
20:1ω7	Paullinic acid	62.3^A^	52.8^A^	18.9^B^	10.1^B^	KW	25.312	3	<0.001	None
20:1ω9	Gondoic acid	24.5^A^	16.2^AB^	6.0^AB^	3.4^B^	KW	15.783	3	0.001	None
20:2ω6	Dihomo-linoleic acid	10.5^A^	8.8^AB^	5.6^AB^	5.0^B^	ANOVA	3.203	3, 35	0.035	Logarithmic
20:3ω9	Mead acid	5.7^A^	6.6^A^	1.6^AB^	1.0^B^	KW	19.508	3	<0.001	None
20:3ω6	Dihomo-γ-linolenic acid	6.6^AB^	11.0^A^	4.7^B^	4.1^B^	ANOVA	5.219	3, 35	0.004	None
20:4ω6	Arachidonic acid	359.9^A^	448.6^A^	217.9^B^	194.4^B^	ANOVA	9.296	3, 35	<0.001	None
20:3ω3	Dihomo-α-linolenic acid	2.2^A^	2.3^A^	0.1^B^	0^B^	KW	26.461	3	<0.001	None
20:4ω3	Eicosatetraenoic acid^a^	3.5^A^	3.6^A^	0.9^AB^	0.2^B^	KW	13.086	3	0.005	None
20:5ω3	Eicosapentaeonic acid	118.2^A^	78.5^AB^	35.0^BC^	15.6^C^	ANOVA	10.597	3, 35	<0.001	Logarithmic
22:0	Behenic acid	54.4^A^	47.2^A^	24.6^B^	17.9^**B**^	KW	26.397	3	<0.001	None
22:4ω6	Docosatetraenoic acid	24.4^AB^	47.4^A^	23.6^B^	21.8^B^	KW	11.366	3	0.010	None
22:5ω3	Clupanodonic acid	85.0^A^	85.0^AB^	40.5^BC^	31.6^C^	KW	18.255	3	<0.001	None
	Other	293.7^A^	258.1^A^	115.3^B^	95.6^B^	KW	25.031	3	<0.001	None
	Sum	8556.7^A^	7621.0^A^	3547.7^B^	2393.4^B^	KW	26.520	3	<0.001	None

(Continued)

**Table 6:** Continued

**Lipid number**	**Fatty acid**	**May (*n* = 12)**	**June (*n* = 8)**	**July (*n* = 10)**	**August (*n* = 9)**	**Test**	**F or H**	**df**	***P***	**Transformation**
	Saturates	2820.2^A^	2609.6^A^	1197.9^B^	747.8^B^	KW	26.446	3	<0.001	None
	Monoenes	4531.4^A^	3739.8^A^	1737.5^B^	1150.2^B^	ANOVA	26.589	3, 35	<0.001	Logarithmic
	PUFA	911.4^A^	1013.5^A^	497.1^B^	399.7^B^	ANOVA	15.805	3, 35	<0.001	Logarithmic
	HUFA	784.6^A^	856.3^A^	434.8^B^	338.3^B^	ANOVA	15.565	3, 35	<0.001	Logarithmic
	Total ω3	368.8^A^	326.4^A^	176.0^AB^	107.3^B^	KW	19.834	3	<0.001	None
	Total ω6	537.0^A^	680.5^A^	319.5^B^	291.4^B^	ANOVA	11.177	3, 35	<0.001	None
	Total ω9	3280.2^A^	2800.9^A^	1337.0^B^	894.5^B^	ANOVA	24.242	3, 35	<0.001	Logarithmic
	AA:EPA	8.33^A^	9.45^AB^	12.17^AB^	22.48^B^	ANOVA	3.369	3, 35	0.029	Logarithmic

## Discussion

This study presents morphometric and blood analyte data of nesting loggerheads from a globally important nesting beach in central eastern Florida and includes routine haematology and plasma biochemistry data, as well as novel blood analytes for this life-stage class (e.g. β-hydroxybutyrate, vitamins, trace nutrients and fatty acid profiles). Specifically, BUN, β-hydroxybutyrate, plasma lipids and fatty acid profiles showed utility as biomarkers of nutritional balance and provided insight into the catabolic state and physiological changes during reproductive activity and energetic demands across nesting seasons. The overall trends and correlations identified in this study revealed insights into phases of fasting and are consistent with the capital breeding strategy of nesting sea turtles as documented in loggerheads nesting on the west coast of Florida (Perrault & Stacy, 2018) and in other species, including leatherbacks (Honarvar *et al.*, 2011; Perrault *et al.*, 2012, 2014, 2016; Plot *et al.*, 2013; Stacy *et al*., 2019), green turtles (Hamann *et al.*, 2002; Hays *et al.*, 2002; Page-Karjian *et al.*, 2020) and hawksbills (*Eretmochelys imbricata*) (Goldberg *et al.*, 2013).

### Morphometrics

Morphometric data from loggerheads nesting at ACNWR were similar in range to a previous study from the central eastern Florida region (Bjorndal *et al.*, 1983) and to other nesting loggerheads along the Atlantic coast of Florida and Georgia (Deem *et al.*, 2009; Flower *et al.*, 2015). However, mean SCL (89.9 cm) of loggerheads from this study was slightly lower than the 37-year historical mean (91.1 cm) of loggerheads nesting on ACNWR. It is notable that the minimum size of sexually mature female loggerheads and green turtles on the ACNWR was observed to have slightly reduced over the past four decades due to population-level effects that are thought to have been associated with demographic and behavioural changes and declines in habitat quality (Phillips *et al.*, 2021). The strong relationship between mass and SCL was expected with larger turtles being heavier, a common observation in turtles of various life-stage classes (Georges & Fossette, 2006; Martins *et al.*, 2022; Perrault *et al.*, 2020; Stacy *et al.*, 2023). The overall mild degree of observed injuries in this study seems representative for nesting turtles (Miller, 1997; Page-Karjian *et al.*, 2020), although partial or even complete flipper amputations are not uncommon in sea turtles (Aoki *et al.*, 2023; Ataman *et al.*, 2021; Deem *et al.*, 2006, Deem *et al.*, 2009; Perrault *et al.*, 2012). The degree of epifaunal fouling (including barnacles, algae and leeches) in this study was similar to findings in a large-scale study of nesting loggerheads from St. George Island, Florida, and juveniles from North Carolina (Ingels *et al.,* 2020; Stamper *et al.*, 2005). Leeches have also been commonly observed on the cloaca and neck of nesting loggerheads and green turtles on Juno Beach, Florida (Loggerhead Marinelife Center, unpublished data). The observed lower epibiota coverage in May compared to July and August suggests nesting turtles presumably decrease activity levels during inter-nesting intervals (Frick *et al.*, 2000) or epibiota growth increases in warmer waters encountered adjacent to nesting beaches.

### Blood analyte data

Given the lack of significant external or behavioural abnormalities, the ACNWR loggerheads can be considered clinically normal representatives of this nesting aggregation in central eastern Florida. This was also reflected in haematology, plasma biochemistry, trace nutrient and vitamin data as reference intervals were mostly within ranges reported for other nesting loggerhead populations in the USA, Canary Islands and Turkey (Deem *et al.*, 2009; Sözbilen *et al.*, 2018; Casal *et al.*, 2009; Flower *et al.*, 2015; Gicking *et al.*, 2004; Molter *et al.*, 2022; Perrault *et al.*, 2016). The only analyte that was in the lower range of reported reference intervals was PCV, a haematological measure of critical clinical importance (Stacy & Innis, 2017). Compared to other studies of nesting loggerheads from the USA, PCV tended to be lower in ACNWR turtles (Deem *et al.*, 2009; Flower *et al.*, 2015). Considerations for this observation include differences in foraging grounds (e.g. dietary composition, abundance) preceding the nesting period, different timing of studies with variable number of study turtles, in migratory distances between foraging and nesting grounds, possibly some degree of underlying chronic but subclinical issues (e.g. from external lesions) although there was no evidence of systemic inflammation or other abnormalities in blood analytes of turtles with <20% PCV (*n* = 3; 15–19% during July and August) or hyporexia resulting in reduced erythropoiesis at the height of the nesting season. Although lymph contamination was not visible during collection of any blood samples, some mild degree that may have affected the PCV cannot definitively be excluded. Total protein as determined by refractometer and Biuret method correlated strongly, similar to other studies in sea turtles (Bolten & Bjorndal, 1992; Rousselet *et al.*, 2013; Perrault *et al.*, 2020).

Fatty acid profiles from blood samples have been documented in wild-captured and cold-stunned juvenile sea turtles, freshwater snapping turtles (Clyde-Brockway *et al*, 2021; Dass *et al.*, 2020; Heniff *et al.*, 2022; Koutsos *et al.*, 2021) and for the first time in nesting sea turtles in this study. The predominant essential fatty acids in nesting turtles included monoenes, ω9 (omega-9), oleic acid 18:1ω9, saturates, palmitic acid 16:0, polyunsaturated fatty acids (PUFA), highly unsaturated fatty acids (HUFA), palmitoleic acid 16:1ω7 and ω6 (omega-6), and also the ratio of arachidonic acid to eicosapentaenoic acid (AA:EPA). Some of these are similar to lipids identified in yolk of green turtles and ostrich eggs, suggesting their involvement in vitellogenesis (Noble *et al.*, 1996; Craven *et al.*, 2008). These fatty acid profiles of nesting loggerheads are substantially different from profiles of foraging neritic juvenile green and Kemp’s ridley sea turtles (*Lepidochelys kempii*), with a predominance of 18:2ω6 and 18:3ω3 in green turtles, but similar concentrations of arachidonic acid 20:4ω6, and higher EPA (20:5ω3) in Kemp’s ridleys (Koutsos *et al.*, 2021). None of these fatty acids were predominant in nesting loggerheads and were substantially lower than values for juvenile green and Kemp’s ridley turtles, in which the predominant monoenes were not detected. The variable observations of plasma lipids across sea turtle studies suggest differences in diet due to species, geographical locations, season and/or life-stage classes, the need for certain circulating fatty acids necessary for vitellogenesis, various stages of fasting or hyporexia with consequent release of different components of fatty acids and components from tissues and/or changes to the primary tissues of origin (e.g. liver, adipose tissue) during phases of hyporexia (McCue, 2008; Price & Valencak, 2012; Secor & Carey, 2011).

### Correlations of BCI with blood analytes

Copper and iron possibly reflect higher body stores in turtles with higher BCI. Because both elements are mostly bound to plasma proteins in circulation, their blood concentrations are largely determined by plasma protein concentrations. The decreasing trend of circulating iron across nesting season suggests utilization of retained and recycled iron stores due to fasting and/or contribution to vitellogenesis (Knutson & Wessling-Resnick, 2003).

### Changes in mass and blood analytes with month of nesting

The observed differences of mass and blood analytes by nesting month confirmed expected trends associated with capital breeding in females across nesting season. Although the data herein do not reflect the same individuals across time, the effect of time appears significant in this cohort of study turtles for several blood analytes. Similar physiological changes have been documented in nesting loggerheads, leatherbacks, green turtles and hawksbills (Hamann *et al.*, 2002; Hays *et al.*, 2002; Perrault *et al.*, 2012, 2014, 2016; Honarvar *et al.*, 2011; Perrault & Stacy, 2018; Goldberg *et al.*, 2013; Page-Karjian *et al.*, 2020). During their nesting season, sea turtles apparently forage little to none (Hopkins-Murphy *et al.*, 2003) and concurrently have high energetic demands (e.g. for mating, nesting/crawling activities, inter-nesting movements, vitellogenesis). This is reflected in the decline in mass across month of nesting season, which has also been observed in nesting leatherbacks, green turtles and hawksbills (Hays *et al.*, 2002; Santos *et al.*, 2010; Goldberg *et al*., 2013; Plot *et al.*, 2013). Although mass declined across nesting season, BCI tended to be lower in August without statistical significance. BCI, a calculated parameter, is considered an important indicator for overall and nutritional assessment in sea turtles (Bjorndal *et al.*, 1983); this parameter may not similarly represent nutritional health in fasting, reproductive adult female turtles in visually adequate body condition. Thus, all available morphometric data should be considered when assessing sea turtles of various life-stage classes.

PCV tended to decrease as nesting seasons progressed, with turtles nesting in May showing the highest PCV and turtles nesting in August showing the lowest. Similar seasonal PCV reduction is seen in leatherbacks and is considered an important indicator of foraging status during nesting season, of remigration interval and potentially, of age (Perrault *et al.*, 2016; Honarvar *et al.*, 2011). Other indicator declines with nesting season progression were related to nutritional and energy metabolism, catabolic state and needs for vitellogenesis in ACNWR loggerheads including cholesterol, triglycerides, calcium, phosphorus, total protein (TP-R and TP-B), albumin, total globulins, iron, alpha-tocopherol, retinol and 23 of 34 fatty acids. Most of these trends were similar to leatherback turtles from Equatorial Guinea and St. Croix, US Virgin Islands and to loggerheads from Casey Key, Florida (Perrault *et al.*, 2014, 2016; Honarvar *et al.*, 2011; Perrault & Stacy, 2018). The observed declines in fatty acids across nesting seasons presumptively reflect utilization of stored energy and progression of fasting state across time, whilst turtles partition energy demands towards egg production and laying. Lipid mobilization prior to and during nesting is expected, but appears to primarily rely on omega-9 fatty acids also seen as most prevalent in wild green turtles (Koutsos *et al.*, 2021), along with use of midchain C:14 myristic acid, as well as C:16 palmitic, the most common saturated fatty acid mobilized into circulation; both are primarily utilized to anchor signalling proteins in cell membranes (Voet & Voet, 2010).

The variability in BHB data from nesting loggerheads indicates phases of fasting and increased lipid utilization across nesting season, as further supported by increasing trend in BUN and decreasing cholesterol, triglycerides and fatty acid profiles. The initial phase of nesting is characterized by full body storage of nutrients (i.e. low BHB and BUN concentrations; high concentrations of lipids), followed by the initial phase of fasting/low food intake and use of carbohydrates (i.e. increase in BHB with a stable BUN; lipids tend to decrease due to ketogenesis) and continuing to an advanced stage of mobilization from tissues (i.e. decrease in BHB and lipids; increases in BUN as protein reserves such as muscle tissues start to be catabolized) (Cherel *et al.*, 1988; Plot *et al.*, 2013; Price *et al.*, 2013; Secor & Carey, 2011). The variability in BHB associated with fasting has been documented in other reptile species; BHB decreases with longer periods of fasting (Price *et al.*, 2013). BUN increased significantly in nesting hawksbills and, in a previous study of nesting loggerheads, had no statistically significant correlation (Perrault & Stacy, 2018; Goldberg *et al.*, 2013). This absence of a trend was attributed to the lack of serial sampling of individual turtles throughout nesting season. In nesting leatherbacks, BUN showed an initial decline followed by an increase towards the end of nesting season (Plot *et al.*, 2013). These variations and tendency towards a BUN increase across nesting seasons in various sea turtle species suggest a switch or beginning transition from lipid to protein catabolism at the end of nesting (Plot *et al.*, 2013; Perrault *et al.*, 2014, Perrault & Stacy, 2018). The degree to which these trends in nutritionally relevant blood analytes occur in individual nesting turtles may be associated with age, reproductive history (re-migrant/previous nester vs. neophyte), frequencies of activities involving mating or nesting (e.g. number of clutches), dietary differences from foraging grounds or individual variation in times at which negative energy balance starts over the course of a nesting season (Goldberg *et al.*, 2013; Perrault & Stacy, 2018; Honarvar *et al.*, 2011).

The electrolytes sodium, chloride and potassium showed significant increases across nesting season, similar to the described trends of chloride in nesting loggerheads (Perrault & Stacy, 2018) and sodium and chloride in nesting green turtles (Page-Karjian *et al.*, 2020; Price, 2017). Trends for sodium and potassium were opposite for nesting leatherbacks and hawksbills (Honarvar *et al.*, 2011; Goldberg *et al.*, 2013). These observations may suggest species-specific differences regarding haemodynamic differences from energetic expenses towards the end of nesting season, considering that vitellogenesis requires plasma proteins that also maintain the oncotic pressure within blood vessels, or alterations in salt gland metabolism. Additionally, variability in diet, water consumption and energetic expenses with migration and reproductive output may also contribute to species variations (Goldberg *et al.*, 2013; Perrault & Stacy, 2018; Plot *et al.*, 2013; Price *et al.*, 2019).

The decline of α-tocopherol suggests its utilization (e.g. for vitellogenesis) and/or release from adipose tissue, respectively. The presence of γ-tocopherol in circulation is associated with increased nitrogen-based antioxidant function against inflammation; however, the concentrations seen in these sea turtles appear minimal (<1% of α-tocopherol) (Ford *et al.,* 2006). It is also possible that plasma α-tocopherol decreases also reflect the high level of mobilization of selenium in vitellogenesis as one has a sparing effect on the other (Combs & McClung, 2017; Guirlet *et al.*, 2008). Vitamin A, as retinol, also declined across nesting season, indicating utilization for vitellogenesis, reproduction and modulation of systemic immune health during fasting. Retinol maintains itself in circulation through liver stores, with usage generally being masked until marked deficiency.

The declines in tissue enzymes amylase and lipase by month do not seem biologically relevant as their means stayed within expected ranges for loggerheads of other life-stage classes (Casal *et al.*, 2009; Deem *et al.*, 2009; Kelly *et al.*, 2015). Zinc also decreased across nesting season, possibly due to utilization or individual hormonal variations across nesting seasons, considering that individual turtles were not repeatedly sampled.

## Conclusions

This study documents morphometric and blood analyte data in nesting loggerheads at ACNWR, a beach that is considered critical for the Northwest Atlantic loggerhead population (Weishampel *et al.*, 2003; Ehrhart *et al.*, 2014). The data herein provide insight into understanding the metabolic and energetic demands in reproductively active female sea turtles and will be useful for spatiotemporal comparisons. Further, we identified biomarkers of nutritional balance (e.g. BUN, β-hydroxybutyrate, plasma lipids cholesterol and triglycerides, calcium, iron, vitamins A and E and fatty acid profiles) for assessing foraging status and phases of reproduction. These biomarkers help identify the catabolic state and thus support the hypothesis of capital breeding in nesting loggerheads. Applications for this information include assessment of individual animals in rescue or managed care settings, comparative study of local, regional, and global intra- or inter-species relationships, and assessment of informed recovery and management strategies.

## Supplementary Material

Web_Material_coae064

## Data Availability

The raw datasets are available from the authors upon request.
