# Supplementary material for: Health assessment of nesting loggerhead sea turtles (Caretta caretta) in one of their largest rookeries (central eastern Florida coast, USA)
Source: Conserv Physiol. 2024 Sep 20;12(1):coae064. doi: 10.1093/conphys/coae064 (PMC11415931; doi:10.1093/conphys/coae064)
Supplement: Web_Material_coae064 [file web_material_coae064.zip › Supplementary_file (1).pdf]

SUPPLEMENTARY TABLES

Supplemental Table 1. Measures of central tendency, range, and reference intervals (with 90% confidence intervals for upper and lower limits) for hematologic and plasma biochemical data in conventional units for nesting loggerhead sea turtles (*Caretta caretta*) from the Archie Carr National Wildlife Refuge. Parametric methods for sample sizes ≥20 but <120 were used to calculate reference intervals, unless otherwise indicated by footnotes. Normality was assessed using the Shapiro-Wilk test, while outliers were detected using the Dixon-Reed test. All plasma samples had hemolysis and lipemia scores ≤1+, which is not considered to cause interference using dry chemistry analysis. Reference intervals could not be calculated for some variables due low sample sizes (<20) or because the majority of the values fell below the detection limit (i.e. right skewed data).

Analyte	Mean±SD	Median	Range	*N*	RI	LRL 90% CI	URL 90% CI	Data distribution, RI method, transformation
Hematology								
Packed cell volume [%]	27±6	28	15–38	25	15–39	11–18	35–42	G, P, N
Immature RBC/100 mature RBC	0	0	0	38	–	–	–	–
White blood cells [/µL]	6670±2150	6100	3900–12700	38	3570–11390	3110–4090	9920–13070	G, P, L
Heterophils [/µL]	3150±1250	2900	1100–6200	33	1340–6340	1100–1640	5200–7730	G, P, L
Immature heterophils [/µL]	10±20	0	0–70	33	–	–	–	–
Total heterophils [/µL]	3160±1250	2900	1100–6200	33	1350–6380	1100–1640	5230–7780	G, P, L
Lymphocytes [/µL]	2530±860	2400	1200–5500[a]	33	1100–3770	750–1440	3430–4120	G, P, N
Monocytes [/µL]	470±230	430	120–1300	33	170–1050	140–220	840–1320	G, P, L
Eosinophils [/µL]	690±600	500	90–2300	33	80–2730	50–130	1750–4260	G, P, L
Basophils [/µL]	30±60	0	0–190	33	–	–	–	–
Heterophil:lymphocyte	1.36±0.80	1.15	0.41–5.00[b]	33	0.34–2.16	0.11–0.58	1.92–2.39	G, P, N
Biochemistry								
Alkaline phosphatase [U/L]	–	<10	<10–40[c]	43	–	–	–	–
Amylase [U/L]	529±197	526	137–1126	43	142–916	55–228	829–1002	G, P, N
Aspartate aminotransferase [U/L]	198±91	176	112–580	43	117–449	110–125	316–944	G, P, B
Blood urea nitrogen [mg/dL]	12.0±5.3	10.9	3.1–28.0	43	4.8–24.4	3.9–5.9	20.7–28.6	G, P, B
Creatine phosphokinase [U/L]	834±768	693	229–5183[d]	43	234–1773	186–294	1410–2229	G, P, L
Calcium [mg/dL]	11.2±4.4	10.8	4.8–31.7	41	5.2–19.2	4.4–6.8	15.2–23.3	NG, R, L
Phosphorus [mg/dL]	9.0±2.2	8.7	5.3–17.3[e]	43	5.6–11.8	5.0–6.2	11.2–12.4	G, P, N
Calcium:phosphorus ratio	1.25±0.30	1.24	0.58–2.07	41	0.67–1.83	0.53–0.80	1.69–1.96	G, P, N
Chloride [mEq/L]	118±6	117	106–134	43	106–131	103–109	128–133	G, P, N
Cholesterol [mg/dL]	189±62	178	97–321	43	97–332	85–112	290–382	G, P, L
Gamma glutamyl-transferase [U/L]	–	<5	<5–10	43	–	–	–	–
Glucose DCA [mg/dL]	79.3±16.2	79.3	39.6–115.3	43	48.7–109.9	41.4–55.9	102.7–117.1	G, P, N
Glucose glucometer [mg/dL]	77.5±12.6	79.3	54.1–93.7	12	–	–	–	–
Lipase [U/L]	–	6	<1–56	43	<1–59	<1	41–89	NG, R, B
Magnesium [mg/dL]	5.8±1.0	2.4	4.4–8.3	43	4.1–7.5	3.7–4.4	7.1–7.8	G, P, N
Potassium [mEq/L]	3.8±0.5	3.6	3.0–5.2	43	2.9–4.8	2.7–3.1	4.5–5.1	G, P, L
Sodium [mEq/L]	144±4	144	133–152	43	135–152	133–137	150–154	G, P, N

Triglycerides [mg/dL]	549±469	513	62–1646	43	35–3655	27–62	2345–4867	NG, R, L
Uric acid [mg/dL]	–	0.67	<0.10–0.99	43	0.17–1.01	<0.10–0.17	0.84–1.01	G, P, N

Abbreviations: B, Box-Cox transformation; CI, confidence interval; G, Gaussian distribution; L, logarithmic transformation; LRL, lower reference limit; N, no transformation; NG, non-Gaussian distribution; P, parametric method; R, robust method; RI, reference interval; SD, standard deviation; URL, upper reference limit.

[a] 5500 cells/μL was an outlier and was removed from reference interval calculations. The second highest value was 3.70 x 10^3 cells/μL.

[b] 5.00 was an outlier and was removed from reference interval calculations. The second highest value was 2.32.

[c] 40 U/L was an outlier. The second highest value was 28 U/L.

[d] 5183 U/L was an outlier and was removed from reference interval calculations. The second highest value was 1565 U/L.

[e] 17.3 mg/dL was an outlier and was removed from reference interval calculations. The next highest value was 4.0 mmol/L.

Supplemental Table 2. Measures of central tendency, range, and reference intervals (with 90% confidence intervals for upper and lower limits) for plasma proteins, trace nutrients, vitamins, and beta hydroxybutyrate in conventional units for nesting loggerhead sea turtles (*Caretta caretta*) from the Archie Carr National Wildlife Refuge. Parametric methods for sample sizes ≥20 but <120 were used to calculate reference intervals, unless otherwise indicated by footnotes. Normality was assessed using the Shapiro-Wilk test, while outliers were detected using the Dixon-Reed test. All plasma samples had hemolysis and lipemia scores ≤1+, which is not considered to cause interference using dry chemistry analysis. Reference intervals could not be calculated for some variables due low sample sizes (<20) or because the majority of the values fell below the detection limit (i.e. right skewed data).

Analyte	Mean±SD	Median	Range	*N*	RI	LRL 90% CI	URL 90% CI	Data distribution, RI method, transformation
Plasma proteins								
Total protein-B [g/dL]	4.4±1.0	4.3	2.9–7.1	39	2.4–6.4	2.0–2.9	5.9–6.8	G, P, N
Total protein-R [g/dL]	4.9±1.6	4.6	2.9–9.3	25	2.6–8.6	2.2–3.1	7.2–10.3	G, P, L
Albumin [g/L]	0.96±0.22	0.93	0.63–1.45	39	0.54–1.38	0.44–0.64	1.28–1.48	G, P, N
Globulins [g/dL]	3.30±0.86	3.23	2.08–5.69	39	1.62–4.99	1.22–2.01	4.59–5.38	G, P, N
Albumin:globulin	0.34±0.07	0.34	0.22–0.49	39	0.21–0.48	0.18–0.24	0.45–0.51	G, P, N
Fibrinogen [g/dL]	0.14±0.18	0.10	0–0.50	18	–	–	–	–
Trace nutrients								
Cobalt [ng/mL]	1.90±3.46	0.68	0.16–16.95	37	0.07–6.87	0.04–0.13	3.47–12.90	NG, R, L
Copper [µg/mL]	0.43±0.13	0.38	0.29–0.84	37	0.09–0.67	0.01–0.19	0.57–0.76	NG, R, N
Iron [µg/dL]	50±28	43	16–145	37	16–122	12–20	95–156	G, P, L
Manganese [ng/mL]	19.8±19.2	15.0	5.7–108.8[a]	37	4.6–45.8	3.4–6.1	34.5–60.7	G, P, L
Molybdenium [ng/mL]	5.6±4.1	4.4	1.2–24.2[b]	37	1.7–11.9	1.4–2.2	9.4–15.0	G, P, L
Selenium [ng/mL]	332±228	241	102–1176	37	87–882	66–115	667–1166	G, P, L
Zinc [µg/mL]	1.17±0.47	1.05	0.72–3.45[c]	37	0.70–1.67	0.63–0.78	1.50–1.86	G, P, L
Vitamins								
α-tocopherol [µg/mL]	16.1±44.1	2.9	0.2–250.0	34	0.1–114.1	0.03–0.2	45.7–284.9	G, P, L
γ-tocopherol [µg/mL]	–	BDL	BDL–1.82[d]	34	–	–	–	–
δ-tocopherol [µg/mL]	–	BDL	BDL–0.15	34	–	–	–	–
Retinol [ng/mL]	622±764	337	167–3070	34	68–1665	46–120	931–2700	NG, R, L
β-hydroxybutyrate								
β-hydroxybutyrate [mmol/L]	1.36±0.77	1.27	0.30–3.86	37	0.41–3.39	0.32–0.53	2.63–4.36	G, P, L

Abbreviations: B, Box-Cox transformation; BDL, below limits of detection; CI, confidence interval; G, Gaussian distribution; L, logarithmic transformation; LRL, lower reference limit; N, no transformation; NG, non-Gaussian distribution; P, parametric method; R, robust method; RI, reference interval; SD, standard deviation; URL, upper reference limit.

[a] 108.8 ng/mL was an outlier and was removed from reference interval calculations. The second highest value was 55.8 ng/mL.

[b] 24.2 was an outlier and was removed from reference interval calculations. The second highest value was 13.5 ng/mL.

[c] 3.45 µg/mL was an outlier and was removed from reference interval calculations. The second highest value was 2.08 µg/mL.

[d] 1.82 µg/mL was an outlier and was removed from reference interval calculations. The second highest value was 0.41 µg/mL.
